# Sexual, Romantic, and Community Experiences of Individuals at the Intersection of Autism and Asexuality

**DOI:** 10.1007/s10508-025-03170-x

**Published:** 2025-06-13

**Authors:** Randolph C. H. Chan, Fei Nga Hung

**Affiliations:** Department of Social Work, The Chinese University of Hong Kong, Shatin, NT Hong Kong

**Keywords:** Autism, Asexuality, Romantic relationships, Sexual behaviors, Sexual orientation

## Abstract

The present study investigated the prevalence of autism among individuals identifying on the asexual spectrum and explored the potential differences in sexual, romantic, and community experiences between autistic and non-autistic individuals on the asexual spectrum. The study included a global sample of 10,419 individuals identifying on the asexual spectrum from the 2020 Ace Community Survey. They completed a questionnaire on asexual identification, romantic relationships, sexual behaviors, and community engagement. The results revealed an autism prevalence rate of 6.9% among individuals on the asexual spectrum, which is higher than the prevalence rate in general populations. Autistic individuals had a stronger identification with their sexual orientation than non-autistic counterparts. They were also more likely to disclose their asexual identity, be in a partnered relationship with others on the asexual or aromantic spectrum, and participate in online LGBTQ communities. The results emphasize the necessity for heightened awareness and understanding of the intersection between autism and asexuality among healthcare professionals, social service providers, and educators. The findings have significant implications for inclusive and affirming sexuality education for individuals on the autism spectrum. Such education is crucial in promoting self-acceptance and empowering them to have greater control over their (a)sexual journeys.

**Supplementary Information:**

The online version contains supplementary material available at 10.1007/s10508-025-03170-x.

## Introduction

Asexuality is a distinct sexual orientation that describes individuals who have a persistent lack of sexual attraction to others (Bogaert, 2015). Previous studies have revealed a potential link between autism and asexuality, identifying a higher prevalence of asexuality among autistic individuals compared to neurotypical individuals, whose neurological development and cognitive functioning align with established norms (Bush, 2019; George & Stokes, 2018; Gilmour et al., 2012). However, there is currently limited empirical research specifically examining the prevalence of autism within the asexual population. Additionally, the existing literature lacks a comprehensive understanding of the differences in lived experiences between autistic and non-autistic individuals on the asexual spectrum. Various aspects of life, including asexual identification, romantic relationships, sexual behaviors, and community engagement, remain understudied within this population. To address these gaps, the present study investigates the intersection of autism and asexuality among a global sample of individuals identifying on the asexual spectrum. By systematically exploring this intersection, the study seeks to provide empirical evidence regarding the prevalence of autism within the asexual population and explore the differences in sexual, romantic, and community experiences between autistic and non-autistic individuals on the asexual spectrum.

### Sexual, Romantic, and Community Experiences of Individuals on the Asexual Spectrum

The asexual spectrum is an umbrella term used to describe individuals who experience little or no sexual attraction toward others, with around 1% of the general population identifying on the asexual spectrum (Bogaert, 2015). There is considerable diversity between various identity labels on the asexual spectrum (Chan & Hung, 2024; de Oliveira et al., 2021). Specifically, within this spectrum, asexuality refers to individuals who experience no sexual attraction; graysexuality describes those who may sporadically experience sexual attraction and consider their sexuality to fall into the “gray area” between asexuality and allosexuality; demisexuality refers to those who experience sexual attraction only after establishing a strong emotional bond with another person (Copulsky & Hammack, 2023).

Sexual attraction and romantic attraction are two distinct categories for describing the sexuality and romantic involvement of individuals within and beyond the asexual community (Antonsen et al., 2020; Clark & Zimmerman, 2022). Asexual spectrum identity can be mapped through the continuum of sexual attraction, ranging from never (for asexual individuals) to rare (for graysexual individuals) to context-dependent (for demisexual individuals) (Hille et al., 2020). Romantic attraction, on the other hand, refers to the desire to form a romantic relationship with individuals of specific sexes or genders, such as aromantic, heteroromantic, homoromantic, biromantic, and panromantic (Brotto et al., 2010). Previous studies revealed a significantly lower proportion of individuals on the asexual spectrum reporting concordant sexual and romantic orientations compared to allosexual individuals (Clark & Zimmerman, 2022). As sexual attraction and romantic attraction may be independent of each other, individuals on the asexual spectrum often experience diverse romantic feelings toward others regardless of their level of sexual attraction (Edge & Vonk, 2024; Van Houdenhove et al., 2015). Specifically, Antonsen et al. (2020) found that 74% of individuals on the asexual spectrum reported experiencing romantic attraction. They often desire romantic relationships that prioritize intimacy, companionship, and emotional connection, while excluding sexual involvement (Brotto et al., 2010; Van Houdenhove et al., 2015).

Despite experiencing low or no sexual attraction, sexual behavior is not necessarily absent in the asexual population. Previous studies have found that the vast majority of individuals on the asexual spectrum report engaging in activities they personally define as sex, which include both partnered and solitary sexual behaviors (Brotto et al., 2010; Hille et al., 2020). Nonetheless, individuals on the asexual spectrum generally report less sexual desire, fewer sexual partners, lower frequency of sexual activity, and fewer prior sexual experiences compared to their allosexual counterparts (Brotto et al., 2010; Clark & Zimmerman, 2022; de Oliveira et al., 2021).

The engagement of asexual individuals within the lesbian, gay, bisexual, transgender, and queer (LGBTQ) community is highly diverse and warrants further investigation. While some asexual individuals may find a strong sense of belonging within the LGBTQ community (Mitchell & Hunnicutt, 2019), others may experience exclusion or marginalization due to the community’s emphasis on sexuality and homonormativity (Chan & Leung, 2023). Consequently, some individuals on the asexual spectrum may reach out for asexual-specific support, primarily through online networks such as the Asexual Visibility and Education Network (AVEN) (Gupta, 2017). These networks play a crucial role in validating and legitimatizing their asexual identity and experiences. Given the diverse range of communities (such as LGBTQ or asexual communities), forms of engagement (online or offline), and levels of involvement, it is crucial to explore how individuals on the asexual spectrum navigate and participate in these communities.

### Autism and Its Impact on Sexual and Romantic Relationships

The autism spectrum is a group of neurodevelopmental conditions that can lead to significant social, communication, and behavioral challenges. Approximately 1 in 100 children (around 1%) around the world are diagnosed with autism (Zeidan et al., 2022). Individuals on the autism spectrum may have difficulty with social interaction and communication across multiple contexts and may show restricted and repetitive patterns of behaviors, interests, or activities in the early developmental stage that may cause impairment in psychosocial functioning (American Psychiatric Association, 2013). Nevertheless, it is important to acknowledge that individuals on the autism spectrum can exhibit a wide range of abilities and characteristics. The manifestation of autistic features can vary significantly among individuals, influenced by factors such as age, developmental level, cognitive abilities, and the severity of symptoms (American Psychiatric Association, 2013).

There is a common misconception that individuals on the autism spectrum lack interest in sexual and romantic relationships (Bennett et al., 2018). Contemporary studies indicate that many autistic individuals express a desire for sexuality and romance, and are capable of healthy sexual functioning (Dewinter et al., 2013; Strunz et al., 2017). While interest in relationships may be similar to neurotypical individuals, some autistic individuals may encounter unique challenges, such as fewer opportunities to meet potential partners and increased concerns about future relationships (Hancock et al., 2020). Lower rates of engagement in further education and employment among some autistic individuals can limit opportunities for social interaction and meeting potential partners (Hancock et al., 2020). Some autistic individuals may also experience uncertainty regarding how to find a partner and navigate relationship dynamics, along with concerns about meeting their partner’s needs and expectations (Hancock et al., 2020; Strunz et al., 2017). Additionally, their challenges with social interaction can impact their involvement in online and offline community spaces (Cuthbert, 2017).

### Intersection Between Autism and Asexuality

Intersectionality is a theoretical framework for understanding how multiple social identities (e.g., gender, race, class, sexual orientation, and ability) intersect and influence one another, shaping individuals’ social positions and lived experiences (Crenshaw, 1989). Intersectionality recognizes that human experiences cannot be fully understood through a single identity in isolation, but rather through the complex interplay of multiple social identities (Cole, 2009). In the context of neurodiversity and (a)sexuality, the intersectionality framework provides valuable insights into the unique challenges and experiences faced by individuals who navigate both autism and asexual spectrum identities (Cuthbert, 2017). By considering intersectionality, it is important to recognize that the sexual, romantic, and community experiences of individuals on the asexual spectrum can vary depending on their autism spectrum conditions.

The intersection between neurodiversity and (a)sexuality is a growing field of research. Previous studies have indicated that individuals on the autism spectrum show higher rates of non-heterosexual orientation (e.g., homosexuality, bisexuality, and asexuality) and lower rates of heterosexuality than neurotypical individuals (George & Stokes, 2018; Gilmour et al., 2012). Recent evidence suggests a notable prevalence of asexuality among individuals on the autism spectrum, as well as elevated rates of autism diagnoses among those on the asexual spectrum (Attanasio et al., 2022; Bush et al., 2021). Some studies have found a higher proportion of autistic individuals who identify as asexual compared to their neurotypical counterparts (Bush, 2019; George & Stokes, 2018; Gilmour et al., 2012; Hartmann et al., 2019; Lewis et al., 2021).

The potential link between autism and asexuality is a topic of frequent discussion within some asexual communities, with a significant number of members in the AVEN identifying themselves as “asexual aspies” (referring to being asexual and having Asperger’s syndrome) (Gupta, 2014). Previous studies found that some asexual individuals reported elevated scores on measures of cold/distant, social withdrawal, and socially avoidant personality styles (Brotto et al., 2010; Yule et al., 2013). These characteristics share some similarities with certain autistic traits, including difficulties in social interaction and relationship formation (Attanasio et al., 2022; Bush et al., 2021). Moreover, some characteristics associated with asexuality are observed among individuals on the autism spectrum. Previous studies showed that a greater proportion of autistic individuals indicated no sexual attraction compared to neurotypical individuals (Dewinter et al., 2017; May et al., 2017). They also reported lower sexual awareness, less sexual desire, and fewer sexual behaviors than their neurotypical counterparts (Bush, 2019; Cheak-Zamora et al., 2019).

Nevertheless, the connection between autism and asexuality should be interpreted with caution, as previous findings raise consideration about assessing asexual identification among individuals on the autism spectrum. Ronis et al. (2021) suggested that some autistic individuals may perceive “asexuality” as a stereotypical label associated with autism rather than a sexual orientation. Additionally, some individuals on the autism spectrum may identify as asexual due to limited sexual experience (Ronis et al., 2021). Consequently, it remains unclear whether asexuality in individuals on the autism spectrum is determined by their self-identification within the asexual community or by the social and interpersonal challenges commonly associated with autism. In contrast to previous studies that predominantly focus on the asexual identity and experiences of individuals on the autism spectrum (Attanasio et al., 2022; Bush et al., 2021; Hartmann et al., 2019; Lewis et al., 2021), it is important to consider comparing the sexual, romantic, and community experiences of autistic and non-autistic individuals on the asexual spectrum. This perspective can allow for a more nuanced understanding of the intersection between autism and asexuality, taking into account the diverse range of experiences within the asexual community.

### Aims of the Present Study

Grounded in the intersectionality framework (Cole, 2009; Crenshaw, 1989), the present study aimed to (1) estimate the prevalence of autism among individuals identifying on the asexual spectrum and (2) examine differences in demographic characteristics, asexual identification, romantic relationships, sexual behaviors, and community engagement between autistic and non-autistic individuals on the asexual spectrum.

## Method

### Participants and Procedure

The present study was based on data from the 2020 Ace Community Survey (https://acecommunitysurvey.org/) (Hermann et al., 2022). Targeted and snowball sampling methods were utilized for participant recruitment. The study information was disseminated on major asexual websites (e.g., The Asexual Visibility and Education Network and The Asexual Agenda) and asexuality-themed groups on different social networking sites (e.g., Facebook, Instagram, Twitter, Tumblr, and Reddit). The study was also circulated on non-English asexual websites, and translation guides were made available to assist participants in completing the questionnaire accurately. Interested individuals were asked to provide informed consent before participating in the study. They were informed that their participation was entirely voluntary and they had the autonomy to skip any part of the survey or withdraw from the study at any time without any consequences.

The present study included a global sample of 10,419 individuals on the asexual spectrum. More than two-thirds of the participants identified as asexual (67.3%), followed by graysexual (11.8%), demisexual (10.0%), questioning if asexual/graysexual/demisexual (8.2%), and other identities on the asexual spectrum (e.g., aceflux, aegosexual, akiosexual/lithsexual, apothisexual, and quoisexual) (2.7%). Around 60.0% of them were on the asexual spectrum only, and 40.0% were on both the asexual and aromantic spectrum (see description of spectrum measures below). The participants had a mean age of 24.63 years (*SD* = 6.50). Almost half of them identified as women (48.8%), while 10.7% identified as men and 40.5% identified as non-binary. Approximately 0.7% of the participants were intersex. Most of them were cisgender (85.6%) and 14.4% were transgender. Approximately 12.8% of them were a racial/ethnic minority in their country of residence. More than four-fifths of the participants had completed tertiary education (82.4%), 13.8% had completed secondary education, and 3.9% had less than secondary education. Nearly half of them were employed (47.5%), 31.7% were students, and 18.0% were not in employment or education. Most of the participants lived in high-income countries (91.6%), followed by upper-middle-income countries (6.8%), and lower-middle-income or low-income countries (1.6%). The country income groups were categorized into four clusters using the World Bank (2020) classification.

### Measures

#### Demographic Characteristics

Participants were asked to provide information regarding their age, gender, education level, employment status, and country income group. They also indicated whether they had an intersex condition, identified as transgender, and considered themselves as a racial/ethnic minority in their country of residence.

#### Autism Spectrum

Participants were asked to indicate which conditions applied to them, including autism spectrum conditions. Four response options were available: “Yes**–**professionally diagnosed,” “Yes–self-diagnosed,” “Unsure,” and “No.” Participants who responded “Yes–professionally diagnosed” were classified as autistic or on the autism spectrum, while those who selected any of the other three options were considered non-autistic or not on the autism spectrum. The decision aims to ensure the accuracy of autism diagnosis and avoid overdiagnosis, which could lead to inflated prevalence rates and misrepresent the actual intersection between autism and asexuality. Nonetheless, a sensitivity analysis was conducted, grouping both professionally diagnosed and self-diagnosed individuals as autistic, to examine their potential differences compared to non-autistic individuals.

#### Asexual Identification

Participants were asked to indicate the sexual orientation labels they most closely identified with. The response options included “asexual,” “gray-asexual (or gray-A, graysexual, etc.),” “demisexual,” “questioning if asexual/gray-asexual/demisexual,” and “other.” Additionally, they were asked to assess the level of strength with which they identified with the selected label. The item was rated on a 5-point scale ranging from 0 *(not strongly at all)* to 4 *(very strongly)*, with higher scores indicating stronger asexual identification.

Participants were asked to indicate the extent to which they were “out” about being asexual or on the asexual spectrum to various groups. These groups included partners, ex-partners, parents, other family members, people in their household, LGBTQIA+ friends, non-LGBTQIA+ friends, classmates, teachers and school staff, coworkers, counselors, and medical professionals. Five response options were provided: “none,” “a few,” “most,” “all,” and “N/A (i.e., not applicable).” The responses to each item were averaged to create an overall score of outness, with higher scores representing higher degrees of outness. Responses of not applicable were not included in the analysis.

Additionally, participants were requested to report their age of asexual awareness (i.e., “At what age did you first start questioning or considering the possibility that you might have the sexual orientation with which you currently identify?”), age of asexual identification (i.e., “At what age did you first identify privately or otherwise with the sexual orientation with which you currently identify?”), and age of coming out (i.e., “At what age did you first tell someone about your current sexual orientation?”).

#### Romantic Relationships

Participants were asked to indicate whether they identified themselves as being on the aromantic spectrum (e.g., aromantics, gray-romantics, demiromantics, aros, etc.). Three response options were available: “Yes,” “No,” and “Questioning/Unsure.” Those who answered “Yes” to this question were considered as identifying themselves as aromantic.

In addition, participants were asked whether they had been in an intimate relationship (“Have you ever had a partnered or intimate relationship?”), whether they had been in an intimate relationship with individuals on the asexual spectrum (“Have you ever had any kind of partnered or intimate relationship with someone you know was asexual or asexual-spectrum?”), and whether they had been in an intimate relationship with individuals on the aromantic spectrum (“Have you ever had a partnered or intimate relationship with someone you know was aromantic or aromantic-spectrum?”). Participants responded to the items on four response options: “Yes, I am in at least one currently,” “Yes, in the past,” “No,” and “Unsure.” Those who answered “Yes, I am in at least one currently” or “Yes, in the past” were considered to have engaged in an intimate relationship.

#### Sexual Behaviors

Participants were asked whether they had engaged in consensual sex (“Have you ever had consensual sex?”), with response options including “Yes,” “No,” and “Unsure.” Those who answered “Yes” to this question were considered to have experienced consensual sex. They were also asked to report their age of sexual debut (“By your best estimate, how old were you at the earliest time you had consensual sex?”).

Additionally, participants were asked to assess the frequency of sexual activity in the past year (“How often have you engaged in consensual sexual activity in the past year?”). Participants responded to the items using seven response options: “not at all,” “1**–**2 times,” “3**–**5 times,” “6**–**10 times,” “11**–**25 times,” “26**–**50 times,” and “at least 50 times.” They were also asked to indicate the level of their sex drive (“How strong is your sex drive/libido, typically?”). The item was rated using five response options, including “nonexistent,” “mild,” “moderate,” “strong,” and “very strong.”

#### Community Engagement

Participants were asked whether they had participated in LGBTQ communities (“Have you ever participated in LGBTQ communities?”). Four response options were available: “Yes**–**both online and offline,” “Yes**–**just online,” “Yes**–**just offline,” and “No.” The responses were then recoded into two variables, i.e., online participation and offline participation. They were also asked to indicate the frequency of engaging in offline asexual groups (“How often do you currently participate in offline asexual groups?”). Six response options were available: “never,” “a few times a year or less,” “once a month,” “a few times a month,” “a few times a week,” and “at least once per day.” The responses to this question were then recoded as a dichotomous scale, where “never” was recoded as “no” and other response options were recoded as “yes,” indicating whether the participants have ever participated in offline asexual groups.

In addition, participants were requested to report their age of participating in the asexual community (“How old were you when you first participated in an asexual community?”). They were asked whether they had met any asexual individuals offline (“Have you ever met someone offline who identified as asexual, gray-asexual, or demisexual, that you know of?”) and whether they had any asexual friends (“Do you have any current friends who identify as asexual, gray -asexual, or demisexual, that you know of?”). Participants responded to these items by choosing one of the three response options: “Yes,” “No,” and “Unsure.” Those who answered “Yes” were considered to have the experience of meeting asexual individuals offline or making asexual friends.

### Data Analysis

Descriptive statistics were used to estimate the prevalence of autism among individuals on the asexual spectrum. Additionally, descriptive statistics were used to provide an overview of the lived experiences of autistic and non-autistic individuals on the asexual spectrum. This included the analysis of demographic characteristics, asexual identification, romantic relationships, sexual behaviors, and community engagement. Before conducting the primary analyses, we assessed our data for normality. The skewness and kurtosis values for continuous variables were within the acceptable range (i.e., skewness < 3.0 and kurtosis < 10.0; Weston & Gore, 2006), indicating no significant violations of normality. Chi-square tests and independent-sample *t*-tests were conducted to examine potential differences in demographic characteristics between autistic and non-autistic individuals on the asexual spectrum. Cramer’s *V* and Cohen’s *d* were used to estimate the effect size for chi-square tests and independent-sample *t*-tests, respectively (Cohen, 1988). Levene’s test for equality of variances was used to assess the assumption of homogeneity of variance across the two groups. In instances where homoscedasticity was violated, the *t* values and their significance were corrected to account for unequal variances.

Multinomial logistic regression was employed to investigate differences in sexual orientation between autistic and non-autistic individuals on the asexual spectrum. Binomial logistic regression and multiple regression were used to explore their differences in asexual identification, romantic relationships, sexual behaviors, and community engagement. The regression analyses controlled for demographic variables, including age, gender, intersex condition, transgender/cisgender identity, racial/ethnic status, education level, employment status, and country income group. To check for potential multicollinearity issues, we examined the tolerance and Variance Inflation Factor (VIF) values across variables. All tolerance and VIF values were within the acceptable range (i.e., tolerance > 0.1 and VIF < 10; Field, 2013), suggesting no multicollinearity issues among the variables.

Sensitivity analyses were conducted to determine whether different grouping methods might affect the study’s outcomes. Specifically, we combined professionally diagnosed and self-diagnosed autistic individuals and re-ran the analysis to estimate their differences in sexual, romantic, and community experiences compared to non-autistic individuals. Additionally, we analyzed the differences between professionally diagnosed and self-diagnosed autistic individuals. All analyses were conducted using SPSS version 28.0.

## Results

### Prevalence of Autism Among Individuals Identifying on the Asexual Spectrum

Of 10,419 individuals identifying on the asexual spectrum, 6.9% of them were professionally diagnosed autism spectrum conditions. Approximately 93.1% of the participants did not receive a professional diagnosis of autism spectrum conditions. This included 9.0% who reported self-diagnosed autism spectrum conditions, 18.1% who were unsure about their autistic status, and 66.0% who did not have an autism spectrum diagnosis.

For the individuals on the autism spectrum, over two-thirds of them identified as asexual (67.6%), followed by graysexual (13.1%), demisexual (9.5%), questioning if asexual/graysexual/demisexual (5.3%), and other identities on the asexual spectrum (4.6%). Among individuals not on the autism spectrum, more than two-thirds of them identified as asexual (67.3%), followed by graysexual (11.7%), demisexual (10.0%), questioning if asexual/graysexual/demisexual (8.4%), and other identities on the asexual spectrum (2.6%).

### Differences Between Autistic and Non-autistic Individuals on the Asexual Spectrum

#### Demographic Characteristics

The results indicated that autistic individuals on the asexual spectrum (*M* = 24.31, *SD* = 6.12) were significantly younger than their non-autistic counterparts (*M* = 24.95, *SD* = 6.89) [*t* (857.40) = − 2.68, *p* = .008, 95% CI − 1.11, − 0.17, Cohen’s *d* = 0.09]. Additionally, there was a significant gender difference between autistic and non-autistic individuals on the asexual spectrum (*χ*^2^[2] = 155.15, *p* < .001, Cramer’s *V* = 0.12). Specifically, more autistic individuals identified as men (15.0%) or non-binary (58.6%) than non-autistic individuals (men: 10.3%, non-binary: 39.2%), whereas fewer autistic individuals (26.4%) identified as women than their non-autistic counterparts (50.5%). Significant differences in intersex status (*χ*^2^[1] = 11.95, *p* = .001, Cramer’s *V* = 0.03) and transgender identity (*χ*^*2*^[1] = 191.34, *p* < .001, Cramer’s *V* = 0.14) were also observed, with more autistic individuals on the asexual spectrum identifying as intersex (1.7% vs. 0.6%) and transgender (31.9% vs. 13.1%) compared to their non-autistic counterparts.

There were also significant differences in education level (*χ*^2^[2] = 35.28, *p* < .001, Cramer’s *V* = 0.06), employment status (*χ*^2^[3] = 114.27, *p* < .001, Cramer’s *V* = 0.11), and country income group (*χ*^2^[2] = 38.70, *p* < .001, Cramer’s *V* = 0.06) between autistic and non-autistic individuals on the asexual spectrum. Specifically, autistic individuals were found to have a higher prevalence of having less than secondary education (7.4% vs. 3.6%) and a lower prevalence of having tertiary education (75.6% vs. 82.9%) compared to their non-autistic counterparts. Individuals on the autism spectrum were more likely to report being unemployed (32.2% vs. 16.9%) and less likely to report being employed (35.8% vs. 48.4%) than their non-autistic counterparts. Additionally, they had a higher likelihood of reporting residence in higher-income countries (97.8%) compared to those not on the autism spectrum (91.1%). Table 1 presents the demographic characteristics of autistic and non-autistic individuals on the asexual spectrum.

**Table 1:** Demographic characteristics of autistic and non-autistic individuals on the asexual spectrum

	Autistic individuals on the asexual spectrum	Non-autistic individuals on the asexual spectrum	Differences	Effect size
	n (%)/M (SD)	n (%)/M (SD)	*χ*^2^/*t*	Cramer’s *V*/Cohen’s* d*
Age	24.31 (6.12)	24.95 (6.89)	− 2.68**	0.09
Gender			155.15***	0.12
Man	108 (15.0%)	1001 (10.3%)		
Woman	190 (26.4%)	4894 (50.5%)		
Non-binary	421 (58.6%)	3799 (39.2%)		
Intersex	12 (1.7%)	57 (0.6%)	11.95**	0.03
Transgender	228 (31.9%)	1262 (13.1%)	191.34***	0.14
Racial/ethnic minorities	94 (13.2%)	1235 (12.8%)	0.08	0.003
Education level			35.28***	0.06
Less than secondary education	53 (7.4%)	346 (3.6%)		
Secondary education	121 (16.9%)	1306 (13.5%)		
Tertiary education	540 (75.6%)	7997 (82.9%)		
Employment status			114.27***	0.11
In employment	256 (35.8%)	4676 (48.4%)		
Student	202 (28.3%)	3092 (32.0%)		
Not in employment or education	230 (32.2%)	1635 (16.9%)		
Other	27 (3.8%)	257 (2.7%)		
Country income group			38.70***	0.06
Lower-middle-income/low-income	1 (0.1%)	162 (1.7%)		
Upper-middle-income	15 (2.1%)	698 (7.2%)		
High-income	702 (97.8%)	8840 (91.1%)		

Data on sex assigned at birth were not collected in the survey https://acecommunitysurvey.org/wp-content/uploads/2021/03/2020rawtext.pdf

*p < .05, **p < .01, ***p < .001

#### Asexual Identification

The results showed that autistic individuals were more likely to identify as other identities on the asexual spectrum (as compared to identifying as asexual) than their non-autistic counterparts (AOR = 1.50, 95% CI 1.01, 2.22). They also reported a stronger asexual identification (*b* = 0.06, *p* = .04, 95% CI 0.003, 0.11), higher levels of outness (*b* = 0.18, *p* < .001, 95% CI 0.12, 0.23), and a younger age of asexual awareness (*b* = − 0.50, *p* = .01, 95% CI − 0.88, − 0.11) than those not on the autism spectrum. However, there were no significant differences in the age of asexual identification (*b* = − 0.23, *p* = .14, 95% CI − 0.55, 0.08) and the age of coming out (*b* = − 0.24, *p* = .09, 95% CI − 0.52, 0.04) between the two groups.

**Table 2:** Asexual identification, romantic relationships, sexual behaviors, and community engagement of autistic and non-autistic individuals on the asexual spectrum

	Autistic individuals on the asexual spectrum	Non-autistic individuals on the asexual spectrum	Differences	Effect size
	n (%)/M (SD)	n (%)/M (SD)	AOR/*b* (SE)	95% CI
Asexual identification				
Sexual orientation				
Asexual	486 (67.6%)	6530 (67.3%)	–	–
Graysexual	94 (13.1%)	1133 (11.7%)	1.15	0.91, 1.46
Demisexual	68 (9.5%)	972 (10.0%)	0.92	0.70, 1.21
Questioning if asexual/graysexual/demisexual	38 (5.3%)	812 (8.4%)	0.81	0.58, 1.15
Other identities on the asexual spectrum	33 (4.6%)	253 (2.6%)	1.50	1.01, 2.22
Strength of asexual identification (0–4)	3.53 (0.65)	3.46 (0.68)	0.06 (0.03)	0.003, 0.11
Outness (1–4)	2.23 (0.77)	1.97 (0.70)	0.18 (0.03)	0.12, 0.23
Age of asexual awareness	16.56 (4.77)	17.89 (5.84)	− 0.50 (0.20)	− 0.88, − 0.11
Age of asexual identification	19.43 (5.57)	20.46 (6.18)	− 0.23 (0.16)	− 0.55, 0.08
Age of coming out	20.05 (5.74)	21.13 (6.25)	− 0.24 (0.14)	− 0.52, 0.04
Romantic relationships				
Aromantic orientation	342 (47.8%)	3812 (39.4%)	1.20	1.03, 1.41
Ever in an intimate relationship	429 (59.7%)	5677 (58.7%)	0.97	0.83, 1.15
Ever in an intimate relationship with individuals on the asexual spectrum	147 (20.7%)	1258 (13.1%)	1.28	1.04, 1.57
Ever in an intimate relationship with individuals on the aromantic spectrum	75 (10.6%)	499 (5.2%)	1.46	1.11, 1.91
Sexual behaviors				
Ever had consensual sex	229 (36.9%)	3477 (39.9%)	0.89	0.74, 1.08
Age of sexual debut	18.99 (3.82)	19.12 (3.61)	0.12 (0.24)	− 0.35, 0.59
Frequency of sexual activity (1–7)	2.77 (1.96)	2.78 (1.97)	− 0.02 (0.13)	− 0.28, 0.24
Sex drive (0–4)	1.52 (1.16)	1.43 (1.01)	0.03 (0.04)	− 0.05, 0.11
Community engagement				
Ever participated in LGBTQ communities				
Online participation	539 (75.4%)	6034 (62.8%)	1.34	1.11, 1.63
Offline participation	356 (49.8%)	3997 (41.6%)	1.10	0.93, 1.30
Ever participated in offline asexual groups	105 (14.7%)	1298 (13.5%)	0.99	0.79, 1.24
Age of participating in the asexual community	20.52 (5.69)	21.97 (6.59)	− 0.48 (0.14)	− 0.75, − 0.21
Ever met any asexual individuals offline	426 (59.3%)	5625 (58.2%)	0.89	0.75, 1.05
Ever had any asexual friends	423 (59.2%)	5101 (52.7%)	1.10	0.93, 1.30

Regression analyses controlled for demographic variables, including age, gender, intersex condition, transgender/cisgender identity, racial/ethnic status, education level, employment status, and country income group

#### Romantic Relationships

The results revealed that autistic individuals were more likely to identify as aromantic (AOR = 1.20, 95% CI 1.03, 1.41), engage in an intimate relationship with other individuals on the asexual spectrum (AOR = 1.28, 95% CI 1.04, 1.57), and engage in an intimate relationship with other individuals on the aromantic spectrum (AOR = 1.46, 95% CI 1.11, 1.91) than their non-autistic counterparts. Nonetheless, there were no significant differences in their experiences of having an intimate relationship (AOR = 0.97, 95% CI 0.83, 1.15).

#### Sexual Behaviors

The results found no significant differences in the experiences of having consensual sex (AOR = 0.89, 95% CI 0.74, 1.08) between autistic and non-autistic individuals on the asexual spectrum. Similarly, there were no significant differences in the age of sexual debut (*b* = 0.12, *p* = .62, 95% CI − 0.35, 0.59), frequency of sexual activity (*b* = − 0.02, *p* = .86, 95% CI − 0.28, 0.24), and level of sex drive (*b* = 0.03, *p* = .43, 95% CI − 0.05, 0.11) between the two groups.

#### Community Engagement

The results revealed that individuals on the autism spectrum were more likely to have engaged in online LGBTQ communities than those not on the autism spectrum (AOR = 1.34, 95% CI 1.11, 1.63). Additionally, they participated in the asexual community at an earlier age than their non-autistic counterparts (*b* = − 0.48, *p* = .001, 95% CI − 0.75, − 0.21). However, no significant differences were found in participation in offline LGBTQ communities (AOR = 1.10, 95% CI 0.93, 1.30), as well as the experiences of engaging in offline asexual groups (AOR = 0.99, 95% CI 0.79, 1.24), meeting asexual individuals offline (AOR = 0.89, 95% CI 0.75, 1.05), and having asexual friends (AOR = 1.10, 95% CI 0.93, 1.30) between the two groups. Table 2 summarizes the asexual identification, romantic relationships, sexual behaviors, and community engagement of autistic and non-autistic individuals on the asexual spectrum.

### Sensitivity Analyses

Professionally diagnosed and self-diagnosed individuals were grouped together as autistic individuals, and regression analyses were conducted to assess their sexual, romantic, and community experiences in comparison to non-autistic individuals. The results from this sensitivity analysis were largely consistent with those reported in the primary analysis (see Table S1 in the online supplementary material). Additionally, the differences between professionally diagnosed and self-diagnosed autistic individuals were analyzed. The results indicated no significant differences between professionally diagnosed and self-diagnosed autistic individuals in romantic relationships, sexual behaviors, and community engagement (see Table S2 in the online supplementary material). However, self-diagnosed autistic individuals were more likely to identify as demisexual (AOR = 0.71, 95% CI 0.51, 0.99) and questioning (AOR = 0.63, 95% CI 0.42, 0.96) (as compared to identifying as asexual) and reported lower levels of outness (*b* = 0.12, *p* = .003, 95% CI 0.04, 0.19) than professionally diagnosed autistic individuals. Sensitivity analyses generally revealed that different grouping approaches did not significantly impact the main results, thereby supporting the robustness of our findings.

## Discussion

The study provides valuable insights into the connections and overlaps between autism and asexuality. Based on a global sample of 10,419 individuals on the asexual spectrum from the 2020 Ace Community Survey, the study found an autism prevalence rate of 6.9% among individuals on the asexual spectrum. The prevalence rate is much higher than the prevalence of autism in general populations (i.e., around 1%; Zeidan et al., 2022). The results provide empirical evidence of the co-occurrence of autism and asexuality. The lack of sexual attraction among individuals on the autism spectrum can be attributed to their distinct patterns of social interaction and communication, which can influence how they navigate intimate relationships (Hancock et al., 2020; Strunz et al., 2017). Some autistic individuals may also have different sensory experiences, which could affect their comfort with physical intimacy and potentially lead to reduced sexual attraction (Attanasio et al., 2022; Bush et al., 2021). Nevertheless, the explanation for the relationship between autism and asexuality is still far from conclusive. More research is needed to explore the biological, psychological, and sociocultural factors that may contribute to the co-occurrence of autism and asexuality in some individuals.

The study identified notable demographic differences between autistic and non-autistic individuals on the asexual spectrum. There were significantly more autistic individuals identifying as men and non-binary compared to their non-autistic counterparts. There were also more intersex and transgender individuals among those on the autism spectrum. The results align with findings from epidemiological studies in allosexual populations, which have observed higher rates of autism among cisgender men (Elsabbagh et al., 2012) and transgender and gender diverse individuals (Warrier et al., 2020). The gender differences observed in the study may stem from inherent disparities in the frequency of autism diagnoses, as cisgender boys have historically been more likely to receive an autism diagnosis compared to cisgender girls (Loomes et al., 2017). Various explanations have also been proposed to account for the higher prevalence of gender variance among individuals on the autism spectrum. Biologically, autism and gender variance may share common genetic patterns and could be influenced by prenatal exposure to testosterone (Dewinter et al., 2017). From a psychosocial perspective, some autistic individuals may face challenges in fitting themselves into social categories, including identifying with a specific gender label (Skorich et al., 2016). Consequently, some may develop more diverse gender identities compared to neurotypical individuals who more readily conform to traditional gender norms (Cooper et al., 2018). Moreover, their reduced awareness of societal norms may foster a sense of liberation from gender expectations and stereotypes, potentially providing space for diverse gender expressions (Stanojević et al., 2021).

Furthermore, the study found that individuals on the autism spectrum were more likely to report having less than secondary education, being unemployed, and living in higher-income countries compared to their non-autistic counterparts. These findings highlight the importance of addressing educational and employment support for autistic individuals (Hancock et al., 2020). An overrepresentation of autistic individuals in higher-income countries may reflect greater access to developmental screening and professional diagnosis in these areas. Future studies should consider relevant social and contextual factors when examining and interpreting demographic disparities in the prevalence of autism diagnosis and asexual identification.

Based on the intersectionality framework (Cole, 2009; Crenshaw, 1989), this study explored the distinct patterns of self-realization, exploration, and disclosure of asexual identity among individuals at the intersection of autism and asexuality. Notably, the results indicated that autistic individuals exhibited a stronger identification with their sexual orientation and were more likely to openly identify as being on the asexual spectrum than non-autistic individuals. They also began questioning or considering asexuality at a younger age. These patterns may be influenced by autistic individuals’ understanding of societal expectations, including those related to sexuality (Bennett et al., 2018; Cooper et al., 2018). This may lead them to explore alternative explanations and identities that align more closely with their experiences and feelings, promoting earlier sexual identity exploration and nurturing a stronger identification with asexuality. Additionally, their direct communication style and reduced sensitivity to social disapproval may have facilitated disclosure and open discussion of their asexuality (Stanojević et al., 2021).

The study provides insights into the romantic relationships of individuals who fall within both the autism and asexual spectrums (Antonsen et al., 2020). The results showed that autistic individuals were more inclined to identify as aromantic than their non-autistic counterparts. This aligns with previous research indicating that some autistic individuals may have different preferences or desires for romantic relationships (Cheak-Zamora et al., 2019; Strunz et al., 2017). These tendencies can be attributed to their challenges in forming emotional connections, which may contribute to a reduced preference for romantic involvement and ultimately lead them to identify as aromantic (Hancock et al., 2020). This study also found that autistic and non-autistic individuals on the asexual spectrum were equally likely to have engaged in a partnered relationship. However, autistic individuals had a higher likelihood of having been in a partnered relationship with others on the asexual spectrum or aromantic spectrum than their non-autistic counterparts. This may be due to the fact that even within the asexual community, autistic individuals may experience varied patterns of romantic attraction when compared to non-autistic individuals. Some individuals on the autism spectrum may have challenges in social interaction and heightened sensitivities to sensory input, which can make some aspects of intimate relationships uncomfortable (Stanojević et al., 2021). The intensity of emotional closeness involved in romantic relationships may be challenging for some autistic individuals, potentially leading them to identify as aromantic or prefer to be in a relationship with someone on the asexual or aromantic spectrum.

Despite differences in asexual identification and romantic relationships, autistic and non-autistic individuals on the asexual spectrum showed similar patterns in their sexual behaviors. Specifically, they had a similar likelihood of having had consensual sex. Their age of sexual debut, frequency of sexual activity, and level of sex drive were not significantly different. The findings suggested that the influence of autism on sexual behaviors might be minimal among individuals on the asexual spectrum.

Furthermore, the study found differences in community engagement between autistic and non-autistic individuals on the asexual spectrum. A larger proportion of autistic individuals had participated in online LGBTQ communities compared with non-autistic individuals. This finding suggests that individuals on the autism spectrum, who may experience difficulties in social interaction and communication, feel more comfortable engaging in LGBTQ communities through online platforms (Cuthbert, 2017). The reduced social demands associated with online interactions may provide a less overwhelming and more controllable environment for them to share experiences and connect with others. Additionally, the findings revealed that autistic individuals participated in the asexuality community at a younger age than their non-autistic counterparts, despite no notable differences in their participation in offline asexual groups or connections with other asexual individuals offline. It is plausible that autistic individuals on the asexual spectrum face significant challenges due to the stigmatization of their dual marginalized identities (Bennett et al., 2018). Therefore, they are eager to seek support and connection within LGBTQ and asexual communities as they navigate the intersections between their autistic and asexual experiences (Gupta, 2017).

### Practical Implications

The findings highlight the need for increased awareness and understanding of the intersection between autism and asexuality among healthcare professionals, social service providers, and educators, so that they have the competence to provide appropriate support and care for individuals across the autism and asexual spectrums. It is important to recognize the unique experiences and needs of autistic individuals on the asexual spectrum, which can inform the development of targeted intervention and support systems to address the needs of this population. Training programs can be provided to caregivers and family members of autistic individuals in order to enhance their understanding of the (a)sexuality of autistic individuals.

The study also has implications for sexuality education for individuals on the autism spectrum. Sexuality education plays a vital role in equipping them with the knowledge and skills to navigate their (a)sexuality, assert their needs, and develop appropriate relationships. It provides an inclusive space for them to discuss their concerns and empowers them to make informed sexual decisions that align with their values and preferences. By validating asexuality as a normal variation of human sexuality, sexuality education addresses the unique needs of autistic individuals who may lack the intuitive understanding of societal norms and expectations related to sexuality. This approach is crucial in fostering self-acceptance and a greater sense of control over their (a)sexual journeys.

### Limitations

Although the present study generates meaningful findings regarding the experiences of individuals across the autism and asexual spectrums, a few limitations should be acknowledged. First, this study underscores the high prevalence of autism among individuals on the asexual spectrum, but it does not attempt to establish or infer the causal relationship between autism and asexuality. Further work is needed to identify the factors that explain the co-occurrence of autism and asexuality. Second, while this study included a large sample of individuals on the asexual spectrum from different parts of the world, the results were derived from a non-probability sample. Participants were self-selected and volunteered to complete the questionnaire, which may introduce sampling bias and affect the representativeness of the findings. Third, there are inherent limitations associated with online surveys, specifically the potential for sampling the same individuals multiple times. This limitation arises due to participant self-selection and the challenges involved in accurately identifying and tracking unique participants. Future work could incorporate additional verification mechanisms (e.g., using a unique identifier system) to minimize the risk of duplicated responses. Fourth, this study found that a substantial proportion of participants reported self-diagnosed autism spectrum conditions or were uncertain about their autistic status, despite not receiving a professional diagnosis. This observation raises important considerations regarding the potential impact of social and systemic barriers on accessing specialized diagnostic services. Factors such as financial constraints, limited availability of qualified healthcare professionals, limited awareness of autism, and the presence of social stigma can hinder individuals from obtaining a formal autism diagnosis. Consequently, these barriers may have contributed to an underestimation of the actual prevalence of autism diagnosis within our sample. Fifth, this study did not capture the manifestation of autistic features. Participants on the autism spectrum may have varying degrees of challenges in their social interaction and communication, which may affect their ability and interest to form romantic, sexual, and social relationships with others. Future studies should take into consideration the influence of autistic features when examining the relationship between autism and asexuality.

### Conclusion

The present study provides empirical evidence of the co-occurrence of autism and asexuality and reveals the differences in demographic characteristics, asexual identification, romantic relationships, and community engagement between autistic and non-autistic individuals on the asexual spectrum. The results found an autism prevalence rate of 6.9% among individuals on the asexual spectrum, which is higher than that among general populations. Autistic individuals on the asexual spectrum had a stronger identification with their sexual orientation and were more open about their asexual identity compared to their non-autistic counterparts. They were also more likely to be in a partnered relationship with others on the asexual or aromantic spectrum and participate in online LGBTQ communities. The exploration of the relationship between autism and asexuality is a promising avenue for future research. By understanding the dynamics and factors underlying the co-occurrence of autism and asexuality, future research may offer insights into the experiences, needs, and challenges faced by autistic individuals on the asexual spectrum, which have significant implications for targeted interventions and support systems for this population. Such research will have the potential to contribute to the current knowledge of human diversity and improve the well-being and inclusion of individuals at the intersection of autism and asexuality.

## Supplementary Information

Below is the link to the electronic supplementary material.

Supplementary file1 (DOCX 38 kb)

## Data Availability

The datasets analyzed in the current study were provided by the Ace Community Survey Team. Requests for access to these data should be directed to the Ace Community Survey Team.
