# Correction: Sexual, Romantic, and Community Experiences of Individuals at the Intersection of Autism and Asexuality

**DOI:** 10.1007/s10508-025-03232-0

**Published:** 2025-07-02

**Authors:** Randolph C. H. Chan, Fei Nga Hung

**Affiliations:** Department of Social Work, The Chinese University of Hong Kong, Shatin, NT Hong Kong

**Correction to: Archives of Sexual Behavior** 10.1007/s10508-025-03170-x

Due to an error during production, the note to Table 1 pertaining to data on sex assigned at birth was incorrect in the PDF version of the article as originally published.

The PDF version of the article has been corrected.

