# Supplementary material for: Sexual, Romantic, and Community Experiences of Individuals at the Intersection of Autism and Asexuality
Source: Arch Sex Behav. 2025 Jun 13;54(6):2199–211. doi: 10.1007/s10508-025-03170-x (PMC12283438; doi:10.1007/s10508-025-03170-x)
Supplement: Supplementary file 1 — Supplementary file1 (DOCX 38 kb) [file 10508_2025_3170_MOESM1_ESM.docx]

Table S1. Asexual identification, romantic relationships, sexual behaviors, and community engagement of autistic and non-autistic individuals on the asexual spectrum

|  | Autistic individuals on the asexual spectrum | Non-autistic individuals on the asexual spectrum | Differences | Effect size |
| --- | --- | --- | --- | --- |
|  | n (%) / M (SD) | n (%) / M (SD) | AOR / *b* (SE) | 95% CI |
| **Asexual identification** |  |  |  |  |
| Sexual orientation |  |  |  |  |
| Asexual | 1066 (64.3%) | 5950 (67.9%) | – | – |
| Graysexual | 216 (13.0%) | 1011 (11.5%) | 1.18 | 0.99, 1.40 |
| Demisexual | 191 (11.5%) | 849 (9.7%) | 1.21 | 1.01, 1.45 |
| Questioning if asexual/graysexual/demisexual | 110 (6.6%) | 740 (8.4%) | 1.08 | 0.87, 1.35 |
| Other identities on the asexual spectrum | 74 (4.5%) | 212 (2.4%) | 1.71 | 1.27, 2.30 |
| Strength of asexual identification (0–4) | 3.52 (0.68) | 3.45 (0.68) | 0.04 (0.02) | -0.001, 0.07 |
| Outness (1–4) | 2.19 (0.77) | 1.95 (0.69) | 0.12 (0.02) | 0.09, 0.16 |
| Age of asexual awareness | 17.29 (5.89) | 17.89 (5.76) | -0.27 (0.14) | -0.55, 0.002 |
| Age of asexual identification | 20.10 (6.25) | 20.44 (6.12) | -0.21 (0.11) | -0.43, 0.01 |
| Age of coming out | 20.75 (6.26) | 21.12 (6.22) | -0.24 (0.10) | -0.44, -0.04 |
| **Romantic relationships** |  |  |  |  |
| Aromantic orientation | 790 (48.0%) | 3364 (38.5%) | 1.20 | 1.07, 1.35 |
| Ever in an intimate relationship | 1035 (62.6%) | 5071 (58.0%) | 1.00 | 0.89, 1.13 |
| Ever in an intimate relationship with individuals on the asexual spectrum | 358 (21.9%) | 1047 (12.1%) | 1.38 | 1.19, 1.60 |
| Ever in an intimate relationship with individuals on the aromantic spectrum | 176 (10.8%) | 398 (4.6%) | 1.52 | 1.24, 1.87 |
| **Sexual behaviors** |  |  |  |  |
| Ever had consensual sex | 605 (41.0%) | 3101 (39.4%) | 0.96 | 0.85, 1.09 |
| Age of sexual debut | 19.25 (4.06) | 19.09 (3.53) | 0.26 (0.16) | -0.06, 0.57 |
| Frequency of sexual activity (1–7) | 2.75 (1.95) | 2.79 (1.97) | -0.05 (0.09) | -0.22, 0.13 |
| Sex drive (0–4) | 1.54 (1.10) | 1.41 (1.00) | 0.09 (0.03) | 0.03, 0.15 |
| **Community engagement** |  |  |  |  |
| Ever participated in LGBTQ communities |  |  |  |  |
| Online participation | 1256 (76.3%) | 5317 (61.3%) | 1.38 | 1.21, 1.58 |
| Offline participation | 826 (50.2%) | 3527 (40.6%) | 1.01 | 0.90, 1.14 |
| Ever participated in offline asexual groups | 232 (14.1%) | 1171 (13.5%) | 0.87 | 0.74, 1.03 |
| Age of participating in the asexual community | 21.40 (6.42) | 21.95 (6.56) | -0.30 (0.10) | -0.50, -0.11 |
| Ever met any asexual individuals offline | 1038 (62.8%) | 5013 (57.4%) | 0.98 | 0.87, 1.11 |
| Ever had any asexual friends | 1024 (62.1%) | 4500 (51.5%) | 1.21 | 1.08, 1.36 |

*Notes*. Professionally diagnosed and self-diagnosed individuals were grouped together as autistic individuals; Regression analyses controlled for demographic variables, including age, gender, intersex condition, transgender/cisgender identity, racial/ethnic status, education level, employment status, and country income group.

Table S2. Asexual identification, romantic relationships, sexual behaviors, and community engagement of professionally diagnosed and self-diagnosed autistic individuals on the asexual spectrum

|  | Professionally diagnosed autistic individuals on the asexual spectrum | Self-diagnosed autistic individuals on the asexual spectrum | Differences | Effect size |
| --- | --- | --- | --- | --- |
|  | n (%) / M (SD) | n (%) / M (SD) | AOR / *b* (SE) | 95% CI |
| **Asexual identification** |  |  |  |  |
| Sexual orientation |  |  |  |  |
| Asexual | 486 (67.6%) | 580 (61.8%) | – | – |
| Graysexual | 94 (13.1%) | 122 (13.0%) | 0.95 | 0.70, 1.29 |
| Demisexual | 68 (9.5%) | 123 (13.1%) | 0.71 | 0.51, 0.99 |
| Questioning if asexual/graysexual/demisexual | 38 (5.3%) | 72 (7.7%) | 0.63 | 0.42, 0.96 |
| Other identities on the asexual spectrum | 33 (4.6%) | 41 (4.4%) | 0.89 | 0.56, 1.44 |
| Strength of asexual identification (0–4) | 3.53 (0.65) | 3.51 (0.70) | 0.05 (0.04) | -0.02, 0.12 |
| Outness (1–4) | 2.23 (0.77) | 2.16 (0.76) | 0.12 (0.04) | 0.04, 0.19 |
| Age of asexual awareness | 16.56 (4.77) | 17.85 (6.56) | -0.42 (0.27) | -0.95, 0.10 |
| Age of asexual identification | 19.43 (5.57) | 20.62 (6.68) | -0.19 (0.22) | -0.62, 0.24 |
| Age of coming out | 20.05 (5.74) | 21.29 (6.58) | -0.14 (0.21) | -0.55, 0.27 |
| **Romantic relationships** |  |  |  |  |
| Aromantic orientation | 342 (47.8%) | 448 (48.1%) | 1.05 | 0.85, 1.29 |
| Ever in an intimate relationship | 429 (59.7%) | 606 (64.8%) | 0.95 | 0.77, 1.18 |
| Ever in an intimate relationship with individuals on the asexual spectrum | 147 (20.7%) | 211 (22.8%) | 0.98 | 0.76, 1.27 |
| Ever in an intimate relationship with individuals on the aromantic spectrum | 75 (10.6%) | 101 (10.9%) | 1.04 | 0.74, 1.46 |
| **Sexual behaviors** |  |  |  |  |
| Ever had consensual sex | 229 (36.9%) | 376 (44.1%) | 0.85 | 0.67, 1.07 |
| Age of sexual debut | 18.99 (3.82) | 19.41 (4.20) | -0.07 (0.33) | -0.72, 0.58 |
| Frequency of sexual activity (1–7) | 2.77 (1.96) | 2.73 (1.95) | 0.08 (0.16) | -0.25, 0.40 |
| Sex drive (0–4) | 1.52 (1.16) | 1.56 (1.05) | -0.06 (0.06) | -0.17, 0.05 |
| **Community engagement** |  |  |  |  |
| Ever participated in LGBTQ communities |  |  |  |  |
| Online participation | 539 (75.4%) | 717 (76.9%) | 1.03 | 0.80, 1.33 |
| Offline participation | 356 (49.8%) | 470 (50.4%) | 1.09 | 0.87, 1.35 |
| Ever participated in offline asexual groups | 105 (14.7%) | 127 (13.7%) | 1.12 | 0.84, 1.51 |
| Age of participating in the asexual community | 20.52 (5.69) | 22.10 (6.88) | -0.37 (0.20) | -0.76, 0.01 |
| Ever met any asexual individuals offline | 426 (59.3%) | 612 (65.5%) | 0.82 | 0.66, 1.03 |
| Ever had any asexual friends | 423 (59.2%) | 601 (64.4%) | 0.88 | 0.71, 1.10 |

*Notes*. Regression analyses controlled for demographic variables, including age, gender, intersex condition, transgender/cisgender identity, racial/ethnic status, education level, employment status, and country income group.
